# Human appropriation of net primary production estimates in the Xinjiang grasslands

**DOI:** 10.1371/journal.pone.0242478

**Published:** 2020-12-02

**Authors:** Xiaotao Huang, Li Ma, Geping Luo, Chunbo Chen, Gangyong Li, Yang Yan, Huakun Zhou, Buqing Yao, Zhen Ma

**Affiliations:** 1 Key Laboratory of Restoration Ecology for Cold Regions Laboratory in Qinghai, Northwest Institute of Plateau Biology, Chinese Academy of Sciences, Xining, Qinghai, China; 2 Key Laboratory of Adaptation and Evolution of Plateau Biota, Chinese Academy of Sciences, Xining, Qinghai, China; 3 University of the Chinese Academy of Sciences, Beijing, China; 4 Xinjiang Institute of Ecology and Geography, Chinese Academy of Sciences, Xinjiang, Urumqi, China; 5 Xinjiang Grassland Technical Popularization Station, Urumqi, China; Michigan State University, UNITED STATES

## Abstract

The human appropriation of net primary production (HANPP) was developed to estimate the intensity of human activities in natural ecosystems, which is still unclear in the Xinjiang grasslands. Using the Biome-Biogeochemical Cycle (Biome-BGC) grazing model in combination with field data, we assessed the HANPP and explored its spatiotemporal patterns in the Xinjiang grasslands. Our results showed that (1) the HANPP increased from 38 g C/m^2^/yr in 1979 to 88 g C/m^2^/yr in 2012, with an average annual increase of 1.47%. The HANPP was 80 g C/m^2^/yr, which represented 51% of the potential net primary production (NPP_pot_), and the HANPP efficiency was 70% in this region. (2) The areas with high HANPP values mainly occurred in northern Xinjiang and northwest of the Tianshan Mountains, while areas with low HANPP values mainly occurred in southern Xinjiang and southwest of the Tianshan Mountains. (3) Interannual variations in HANPP and NPP_pot_ were significantly positively correlated (P<0.01). Interannual variations in HANPP efficiency and grazing intensity were negatively correlated (P<0.01). These results can help identify the complex impacts of human activities on grassland ecosystems and provide basic data for grassland management.

## Introduction

Humans have extensively altered global ecosystems [1,2]. Complex and highly dynamic interrelations exist between human activities and socioeconomic changes [3–5]. To better understand these interrelations, the human appropriation of net primary production (HANPP) has been widely used [6–11]. (The HANPP reflects how strongly humans affect net primary production (NPP) and its availability in ecosystems and has proven to be a useful metric for assessing the environmental impacts of land use at the regional or global scale [10,12].

The HANPP indicator framework has been applied within empirical studies at various spatial and temporal scales. Global estimates of the HANPP have shown an increasing correlation with socioeconomic development, with a global HANPP of 25–30% calculated over the last several decades [7,12–14]. Studies on the HANPP at the continental [11,15–17] and national scales, such as those in Italy [4], New Zealand [18], Bangladesh [19], China [20,21] and West Africa [10], have shown broad interest in quantifying land use and human biomass extraction.

Xinjiang is located in Northwest China, an arid region with widely distributed grassland ecosystems. The grassland area accounts for 34% of the total land area in Xinjiang, and grazing is the main anthropogenic activity in the local grasslands. Due to the arid climate, the grassland ecosystem in Xinjiang is extremely fragile and generally more sensitive to human activity [22]. In addition to the increasing grazing intensity in recent decades, grassland degradation has become widespread in this region [23,24]. These changes have drastically altered the grassland carbon cycling, ecosystem services, and sustainability of the region [10,17]. Quantification of the HANPP can be conducive to further evaluating the effects of grazing on ecosystem productivity, biodiversity, and the capacity of the ecosystem to provide services [25,26]. However, previous studies have not provided clear knowledge in this field in this region, which goes against the sustainable use of grassland resources in Xinjiang.

In this study, the HANPP in the Xinjiang grasslands was estimated using the Biome-Biogeochemical Cycle (Biome-BGC) grazing model in combination with large amounts of field data. This model can effectively estimate total potential net primary production (NPP_pot_) and total actual net primary production (NPP_act_) in grazed grassland; then, HANPP can be estimated. We focused on three objectives: (1) analyze the interannual variations in the HANPP, (2) explore the spatial patterns of the HANPP, and (3) reveal the relationships between the subcomponents of the NPP and grazing intensity in the Xinjiang grasslands from 1979 to 2012.

## Materials and methods

### Study area

**Fig 1:**
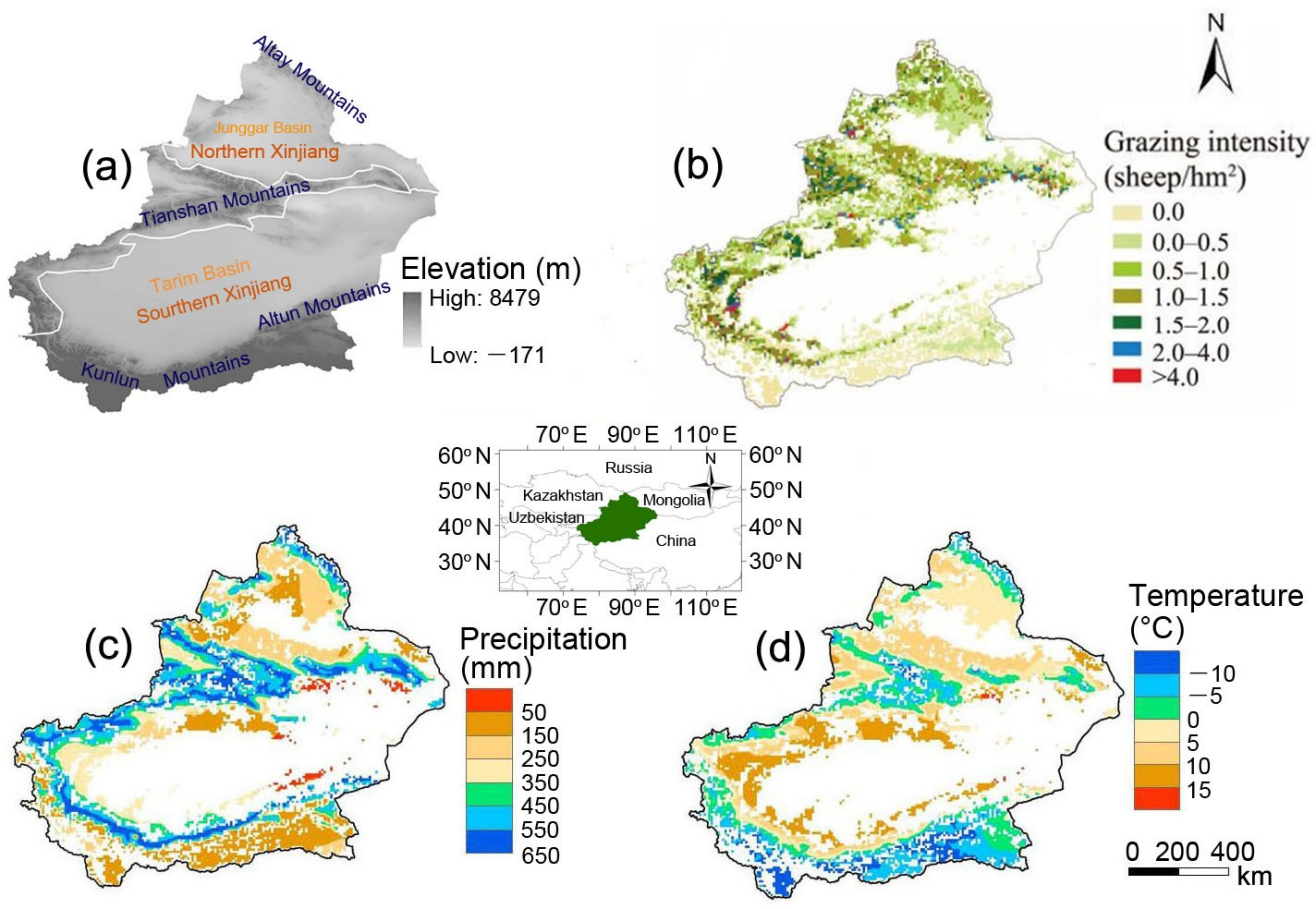
Distribution of elevation (a), grazing intensity (b), precipitation (c) and temperature (d) in the Xinjiang grasslands during 1979–2012 (Republished from Huang et al (2018) under a CC BY license, with permission from Journal of Arid Land, original copyright 2018.).

The Xinjiang Uyghur autonomous region in Northwest China is far from the ocean and covers an area of 1.66×10^6^ km^2^. The Altay Mountains are to the north, the Kunlun Mountains are to the south, the Tianshan Mountains are in the middle, and the vast Junggar Basin and Tarim Basin are almost completely surrounded by these three mountain ranges. Xinjiang has a typical continental arid climate, with limited precipitation and a large daily temperature range. South Xinjiang belongs to a warm temperate zone, and North Xinjiang belongs to a middle temperate zone. The annual mean temperature is 9–12°C, and the mean annual precipitation is 100–200 mm in North Xinjiang and 16–85 mm in South Xinjiang. The ecological environment in Xinjiang is extremely sensitive to climate change and human activities. The total area of the natural grassland is 5.7×10^5^ km^2^, which accounts for 34% of Xinjiang's land area (Fig 1). Most grasslands in Xinjiang are used for grazing, and with the increase in grazing intensity in recent decades, large areas of grasslands have suffered severe deterioration [27,28].

### Methods

Most grasslands in Xinjiang were used for grazing, which is the most important human activity in the region [17,29]. Thus, for simplicity, grazing can be assumed to be the only human activity when estimating HANPP in the Xinjiang grasslands [17,30,31]. The Biome-BGC grazing model includes effective grazing processes; therefore, this model can estimate NPP in both grazed and ungrazed conditions over large areas [23,30]. In the model, total NPP can be estimated as follows [32]:

$$NPP=C_{\mathrm{veg}}^{'}+C_{\mathrm{litter}}+D_{r}$$ (1)

Where C'_veg_ is the vegetative carbon difference between the end of the vegetation period and the start of the vegetation period each year, C_litter_ is the litter carbon, and D_r_ (g C/(ha.d)) is the carbon consumed by domestic animals (NPP harvest) [33].

$$D_{r}=G_{e}S_{r}(C_{\mathrm{leaf}}–{\left(C_{\mathrm{leaf}}\right)}_{U})(0<D_{r}<S_{r}D_{x})$$ (2)

where G_e_ is the grazing efficiency of the domestic livestock (ha/d per sheep unit) which means grazing area in one day per sheep unit, S_r_ is the grazing intensity (sheep/ha); C_leaf_ is the C in the leaf biomass (g C/m^2^); (C_leaf_)_U_ is the residual aboveground C_leaf_ that is unavailable to domestic livestock (g C/m^2^); and D_x_ is the consumption rate of the domestic livestock based on satiation (g C/(d.sheep)). According to Luo et al. [32], the grazing efficiency of domestic livestock is 0.011 ha/d per sheep unit, (C_leaf_)_U_ is 6.75 g C/m^2^ [33,34], and D_x_ is 2.4 kg C/(d.sheep). In this study, HANPP is calculated based on Haberl et al. [7]:

$$HANPP=NPP_{\mathrm{pot}}-NPP_{\mathrm{act}}+D_{r}$$ (3)

where NPP_act_ is the total NPP in grazed conditions (g C/m^2^) and NPP_pot_ is the total NPP in ungrazed conditions (g C/m^2^) (D_r_ = 0). In this study, according to the HANPP efficiency definition by Fetzel et al. [18], the HANPP efficiency is calculated as follows:

$$HANPP\;efficiency=D_{r}/HANPP$$ (4)

A high HANPP efficiency indicates a high ratio of consumption by domestic livestock to the total HANPP and the bulk of the appropriated biomass that enters the socioecological system [15].

### Data

In this study, the required data included the model input data and validated data. The model input data included grazing data, meteorological data, and other auxiliary data. The model-validated data included the observed NPP data. Grasslands in Xinjiang are public spaces open to everyone; therefore, special permission was not required for access.

#### Grazing data

Grazing data at the regional scale were derived from the Food and Agriculture Organization of the United Nations (FAO) (http://www.fao.org/docrep/010/a1259e/a1259e00.htm) by converting the raster data to ASCII text using the R program. To ensure a high degree of data precision, these grazing data were corrected in accordance with the statistics from the Xinjiang yearbook [35]. These grazing data from the Xinjiang yearbook were collected through actual investigations by the government. Grazing data at the model validation points were collected through field investigations. The livestock included cattle, sheep, goats, horses, camels, and yaks. According to the standards provided by the Ministry of Agriculture of the People's Republic of China, one goat equals 0.9 sheep, one cattle equals 6.0 sheep, one horse equals 6.0 sheep, one yak equals 4.5 sheep, and one camel equals 8.0 sheep (http://www.chinaforage.com/standard/zaixuliang.htm). We converted all livestock into sheep units according to these standards.

#### Meteorological data

The meteorological data included the daily maximum and minimum air temperatures, daily average air temperature, humidity, incident solar radiation, precipitation and day length. These data at the regional scale were derived from the China Meteorological Forcing Data set (http://westdc.westgis.ac.cn/data/7a35329c-c53f-4267-aa07-e0037d913a-21) by converting NetCDF data to ASCII text using the R program. These data sets can be used for ecological modeling. The meteorological data at the model validation points were obtained through actual observations.

#### Other auxiliary data

In this study, C3 grass (the default in the model) was used to determine the ecophysiological parameters, and some key parameters, including the yearday to start new growth, yearday to end litterfall, new fine root C:new leaf C and average specific leaf area of the canopy, were corrected according to previous studies in the Xinjiang grasslands [17,32]. The elevation data were derived from the WorldClim database (https://www.worldclim.org). The soil data included sand/silt/clay percentages and the effective soil depth. These data were derived from the Harmonized World Soil Database (HWSD) (http://westdc.westgis.ac.cn/data/611f7d50-b419-4d14-b4dd-4a944b141-175). The soil and elevation data at the model validation points were obtained through field investigations.

#### Model validation data

The model validation data were collected from both field observations and previous publications by Han et al. [23]. There were 67 plots with annual NPP data, including 23 plots under the ungrazed condition and 44 plots under the grazed condition. Due to the difficulty in measuring NPP intake by livestock, all the NPP data collected from the grazed plots represented the NPP remaining in the grassland ecosystems after grazing.

## Results

### Model validation

**Fig 2:**
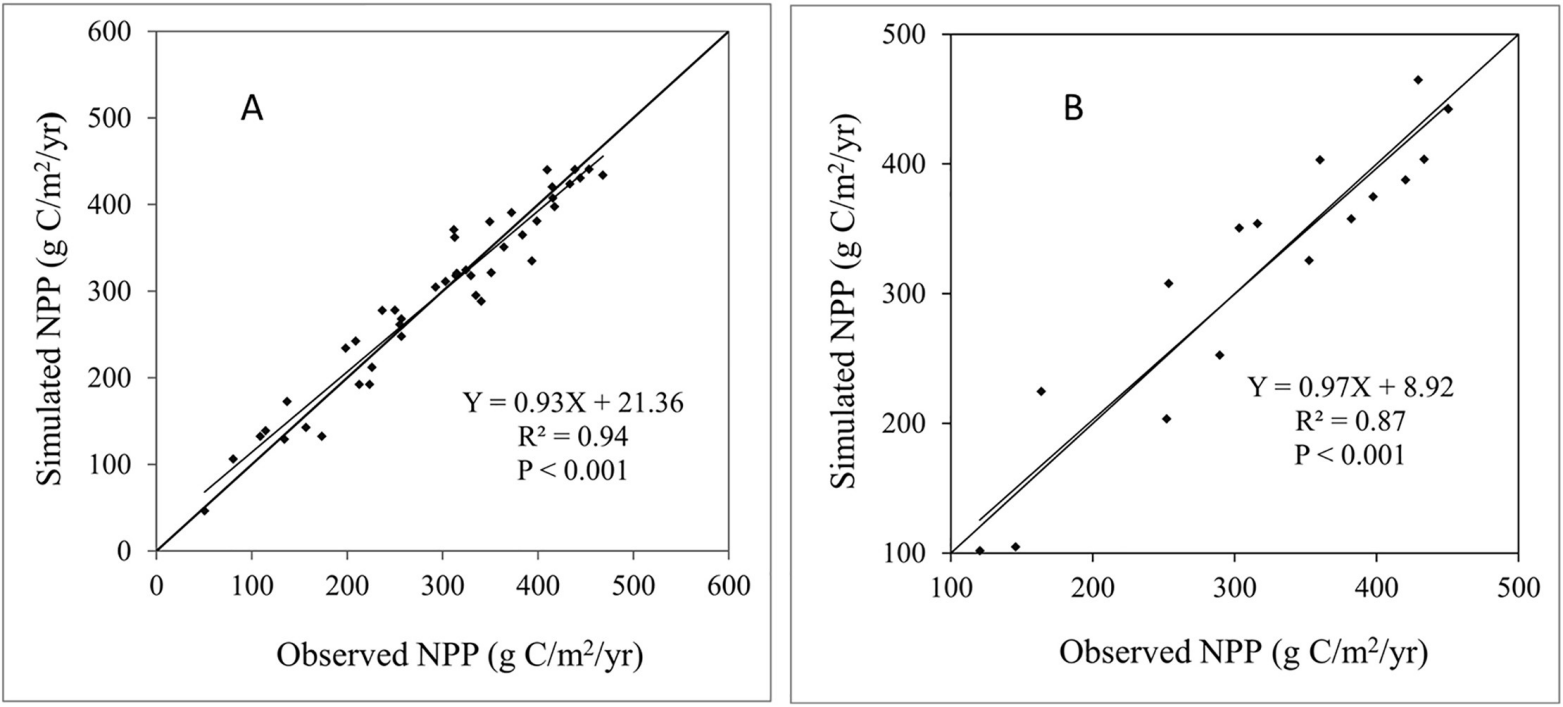
Comparisons of the annual NPP based on simulated and observed field data under grazed (A) and ungrazed (B) conditions (NPP–net primary production).

Previous studies have shown that the Biome-BGC grazing model can effectively estimate the flux and storage of carbon in grassland ecosystems [17]. However, in this study, this model still needs to be validated with respect to the NPP simulation results. We compared NPP estimates of the model with NPP validation data. The results show that the model simulated NPP well under both the grazed (R^2^ = 0.94) and ungrazed (R^2^ = 0.87) conditions (Fig 2).

### Temporal variation in the HANPP in the Xinjiang grasslands

**Fig 3:**
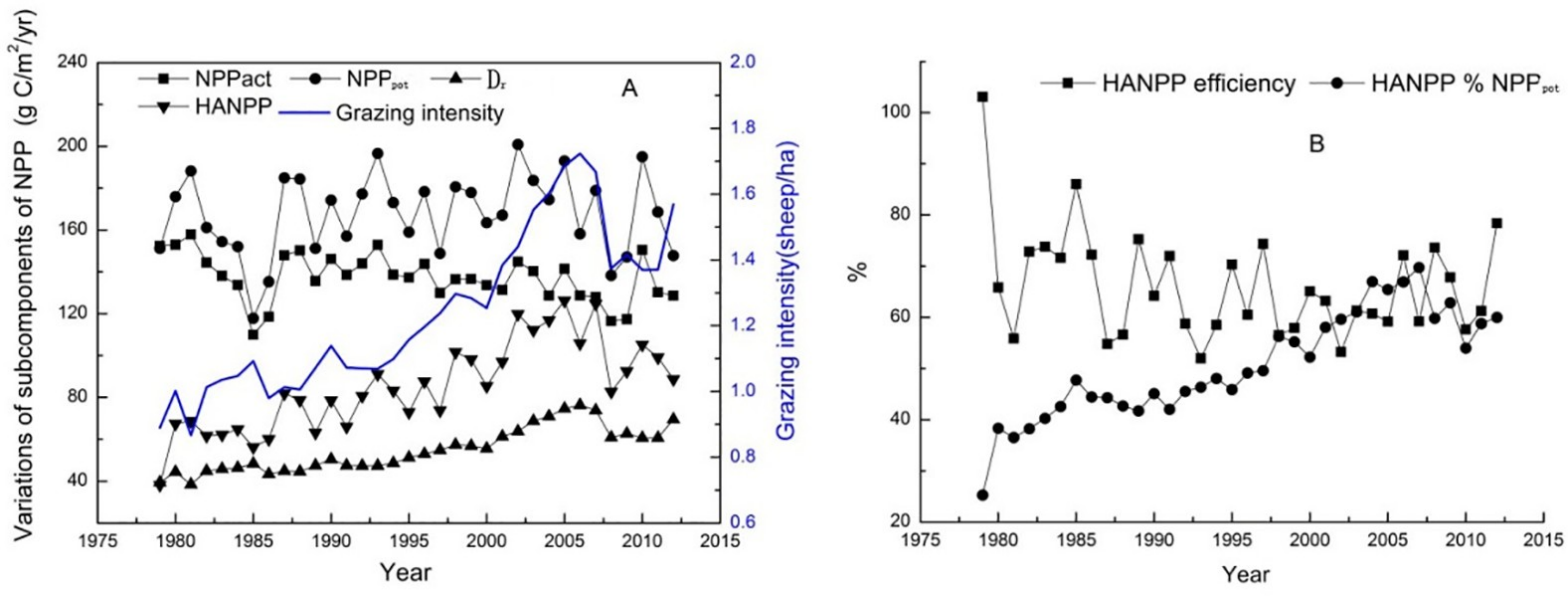
Interannual variations in subcomponents of NPP in the Xinjiang grasslands from 1979 to 2012.

Fig 3A shows the interannual variations in the NPP subcomponents (i.e., HANPP, NPP_pot_, NPP_act_, and D_r_) in the Xinjiang grasslands. The HANPP increased from 38 g C/m2/yr in 1979 to 88 g C/m2/yr in 2012, with an average annual increase of 1.47%. From 1979 to 2005, with increased grazing intensity, the HANPP fluctuated but increased from 38 to 126 g C/m^2^/yr, with an average annual increase of 3.26 g C/m^2^/yr. A similar trend was observed for D_r_, which increased slowly from 1979 to 2005. From 2005 to 2010, the grazing intensity decreased remarkably, and the HANPP noticeably decreased, with an average annual decrease of 3.50 g C/m^2^. In general, with the increase in grazing intensity, the difference between NPP_pot_ and NPP_act_ increased.

Fig 3B shows the interannual variations in HANPP efficiency and HANPP%NPP_pot_ in the Xinjiang grasslands from 1979 to 2012. The HANPP efficiency showed frequent fluctuations and an average annual decrease of 0.74% from 1979 to 2012. In general, D_r_ was the major contributor to the HANPP in the Xinjiang grasslands from 1979 to 2012. HANPP%NPP_pot_ generally showed an increase from 25% to 60%, and the average annual increase was 1.03% from 1979 to 2012. From 1979 to 2005, the changes in HANPP efficiency and HANPP%NPP_pot_ generally showed opposite trends with the increase in grazing intensity.

### Spatial patterns of the HANPP in the Xinjiang grasslands

**Fig 4:**
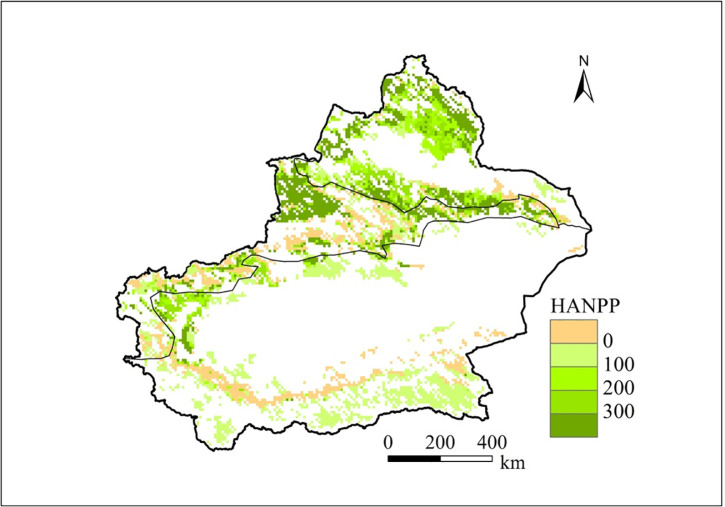
Maps of the average annual HANPP in the Xinjiang grasslands from 1979 to 2012.

Fig 4 displays the spatial distribution of the HANPP in the Xinjiang grasslands. The areas with high HANPP values mainly occurred in northern Xinjiang and northwest of the Tianshan Mountains. The low HANPP values mainly occurred in southern Xinjiang and southwest of the Tianshan Mountains. The map shows that negative HANPP values occurred in some regions that were mainly distributed in the low mountainous areas of the Kunlun Mountains and southwest of the Tianshan Mountains. Our results showed that the HANPP was 80 g C/m^2^/yr, which accounted for 51% of the NPP_pot_ in the Xinjiang grasslands.

### Relationships between the subcomponents of NPP and grazing intensity

Through a pairwise correlation analysis, we found that the correlation coefficient between the interannual variations in HANPP and grazing intensity from 1979 to 2012 was 0.857, and the correlation coefficient between the interannual variations in HANPP and NPP_pot_ from 1979 to 2012 was 0.591 (P<0.01); both correlations were significant according to Pearson's correlation analysis. The correlation coefficient of the interannual variations in the HANPP and HANPP efficiency was -0.655 (P<0.01), indicating a significant negative correlation according to Pearson's correlation analysis. The NPP_act_ and HANPP efficiency were both negatively correlated with grazing intensity, and the correlation coefficients were -0.395 and -0.212, respectively (Table 1). This result indicates that low grazing intensity is more likely to bring a high HANPP efficiency and NPP_act_, while high grazing intensity is more likely to result in a low HANPP efficiency and NPP_act_.

**Table 1:** Correlation coefficients between the subcomponents of NPP and the grazing intensity in the Xinjiang grasslands from 1979 to 2012.

	Grazing intensity (sheep/ha)	HANPP (g C/m^2^/yr)	NPP_pot_ (g C/m^2^/yr)	NPP_act_ (g C/m^2^/yr)	D_r_ (g C/m^2^/yr)	HANPP efficiency (%)	HANPP%NPP_pot_ (%)
Grazing intensity (sheep/ha)	1						
HANPP (g C/m^2^/yr)	0.857**	1					
NPP_pot_ (g C/m^2^/yr)	0.16	0.591**	1				
NPP_act_ (g C/m^2^/yr)	-0.395*	-0.068	0.736**	1			
D_r_ (g C/m^2^/yr)	1.000**	0.857**	0.16	-0.395*	1		
HANPP efficiency (%)	-0.212	-0.655**	-0.807**	-0.340*	-0.212	1	
HANPP%NPP_pot_ (%)	0.951**	0.887**	0.159	-0.498**	0.951**	-0.373*	1

*significant correlation at P<0.05 according to Pearson's correlation analysis

**Extremely significant correlation at P<0.01 according to Pearson's correlation analysis.

## Discussion

### Spatiotemporal variations in the HANPP in the Xinjiang grasslands

With the increased grazing intensity from 1979 to 2005, the HANPP values in the Xinjiang grasslands significantly increased, which indicates that the pressure of grazing on grassland ecosystems has increased. During 2000–2005, the NPP_act_ values of the Xinjiang grasslands were significantly lower than the NPP_pot_ values, indicating that overgrazing occurred in the Xinjiang grasslands during this period. From 2005 to 2010, the HANPP values decreased mainly because of government policies to control the grazing intensity to improve the sustainable utilization of grasslands [36]; thus, there was a temporary decrease in grazing pressure on the grassland ecosystem in Xinjiang. In general, the HANPP increased with the decrease in NPP_act_ as a result of unsustainable livestock grazing in the Xinjiang grasslands during 1979–2012. Due to overgrazing intensity, widespread grassland degradation occurred in Xinjiang [37–39], resulting in large differences between NPP_pot_ and NPP_act_. These differences might explain why high grazing intensity tended to result in low HANPP efficiency and NPP_act_, whereas low grazing intensity had the opposite effects [40,41].

The high HANPP values were mainly distributed in northern Xinjiang and northwest of the Tianshan Mountains, which can mainly be attributed to the increased grazing intensity and good vegetation growth in these grasslands. The regions with low HANPP values were mainly distributed in southern Xinjiang and southwest of the Tianshan Mountains as a result of the relatively low grazing intensity and poor vegetation growth in these regions. In particular, negative HANPP values occurred in some regions, indicating that moderate grazing obviously promoted plant growth in these regions [32]. The spatial distribution of the HANPP values varied among regions due to differences in natural conditions (climate, vegetation growth, etc.) and grazing management in the Xinjiang grasslands. Therefore, we should take corresponding management measures according to the actual situations of the different areas to achieve sustainable use of grassland resources.

### Comparisons with previous studies

The HANPP framework provides a set of integrated socioecological indicators that assess how human activities alter energy flows in ecosystems through land use, and there has been considerable quantitative research on the HANPP at global and regional scales [10–12,14,21]. However, due to the differences in study areas, research methods, etc., the results varied in the different studies. Most of the previous studies did not thoroughly consider grazing processes when estimating the HANPP for grazed grasslands, which resulted in biased results in their studies. Huang et al. [17] estimated the HANPP in Central Asian grasslands using the Biome-BGC grazing model, which included effective grazing processes. In their study, the HANPP was 47 g C/m^2^/yr, which accounted for 34% of the NPP_pot_ in Central Asian grasslands [17]. In our research, we also used the Biome-BGC grazing model. The HANPP was 80 g C/m^2^/yr in the Xinjiang grasslands, which was higher than the HANPP level in Central Asian grasslands, and the value accounted for 51% of the NPP_pot_, which was significantly higher than that in the Central Asian grasslands. A large difference occurred in the overall patterns of grassland HANPP between Xinjiang and Central Asia because of the different national conditions. Although the same method was used as in previous studies, two advantages were observed in our study. First, compared with the results of Huang et al. [17], the results of our study were calibrated and validated via more field data, enhancing the reliability of the results. Second, the higher resolution (10 * 10 km) in our study than in the study by Huang [17] in Central Asian grasslands (40 * 40 km) yielded more detailed information. This study provided a more detailed and reliable theoretical reference to formulate reasonable grazing management practices in the Xinjiang grasslands. Moreover, as information to date remains limited, we encourage additional studies in this region to verify our results and identify a safe threshold of HANPP.

## Conclusions

The Biome-BGC grazing model was used in combination with field data to estimate the HANPP and explore its spatiotemporal patterns in the Xinjiang grasslands. Our results showed that HANPP generally increased from 1979 to 2012 due to increased grazing intensity in the Xinjiang grasslands. The HANPP was 80 g C/m^2^/yr, which represented 51% of the NPP_pot_ in the Xinjiang grasslands, and the HANPP efficiency was 70% in this region. The HANPP and HANPP%NPP_pot_ were greatly affected by grazing intensity. The HANPP showed strong regional differences due to the differences in natural conditions (climate, vegetation growth, etc.) and grazing management in the Xinjiang grasslands. The high HANPP values mainly occurred in northern Xinjiang and northwest of the Tianshan Mountains due to the high grazing intensity and good plant growth in these regions. Low HANPP values mainly occurred in southern Xinjiang and southwest of the Tianshan Mountains due to low grazing intensity and poor plant growth. Negative HANPP values occurred in some regions, indicating that moderate grazing promoted plant growth in these regions. This research provides a better understanding of the spatiotemporal patterns of the HANPP in the Xinjiang grasslands and offers a more detailed and reliable theoretical reference to formulate reasonable grazing management in different regions.

## References

[1] Sanderson EW, Jaiteh M, Levy MA, Redford KH, Wannebo AV, et al (2002) The human footprint and the last of the wild. Bioscience 52: 891–904.

[2] Ellis EC, Ramankutty N (2008) Putting people in the map: anthropogenic biomes of the world. Frontiers in Ecology and the Environment 6: 439–447.

[3] Haberl H, Erb KH, Krausmann F, Adensam H, Schulz NB (2003) Land-use change and socio-economic metabolism in Austria—Part II: land-use scenarios for 2020. Land Use Policy 20: 21–39.

[4] Niedertscheider M, Kuemmerle T, Mueller D, Erb K-H (2014) Exploring the effects of drastic institutional and socio-economic changes on land system dynamics in Germany between 1883 and 2007. Global Environmental Change-Human and Policy Dimensions 28: 98–108. 10.1016/j.gloenvcha.2014.06.006 25844027 PMC4375829

[5] Guo S, Yang G, Li Q, Zhao C (2015) Observation and estimation of the evapotranspiration of alpine meadow in the upper reaches of the Aksu River, Xinjiang. Journal of Glaciology and Geocryology 37: 241–248.

[6] Haberl H (1997) Human appropriation of net primary production as an environmental indicator: Implications for sustainable development. Ambio 26: 143–146.

[7] Haberl H, Erb KH, Krausmann F, Gaube V, Bondeau A, et al (2007) Quantifying and mapping the human appropriation of net primary production in earth's terrestrial ecosystems. Proceedings of the National Academy of Sciences of the United States of America 104: 12942–12945. 10.1073/pnas.0704243104 17616580 PMC1911196

[8] Haberl H, Erb K-H, Krausmann F (2014) Human appropriation of net primary production: ptterns, trends, and planetary boundaries. In: Gadgil A, Liverman DM, editors. Annual Review of Environment and Resources, Vol 39 pp. 363–391.

[9] Ma T, Zhou C, Pei T (2012) Simulating and estimating tempo-spatial patterns in global human appropriation of net primary production (HANPP): A consumption-based approach. Ecological Indicators 23: 660–667.

[10] Morel AC, Sasu MA, Adu-Bredu S, Quaye M, Moore C, et al (2019) Carbon dynamics, net primary productivity and human-appropriated net primary productivity across a forest-cocoa farm landscape in West Africa. Global Change Biology 25: 2661–2677. 10.1111/gcb.14661 31006150

[11] Zhang Y, Pan Y, Zhang X, Wu J, Yu C, et al (2018) Patterns and dynamics of the human appropriation of net primary production and its components in Tibet. Journal of Environmental Management 210: 280–289. 10.1016/j.jenvman.2018.01.039 29407188

[12] Krausmann F, Erb K-H, Gingrich S, Haberl H, Bondeau A, et al (2013) Global human appropriation of net primary production doubled in the 20th century. Proceedings of the National Academy of Sciences of the United States of America 110: 10324–10329. 10.1073/pnas.1211349110 23733940 PMC3690849

[13] Vitousek PM, Ehrlich PR, Ehrlich AH, Matson PA (1986) Human appropriation of the products of photosynthesis. Bioscience 36: 368–373.

[14] Zhou C, Elshkaki A, Graedel TE (2018) Global Human appropriation of net primary production and associated resource decoupling: 2010–2050. Environmental Science & Technology 52: 1208–1215. 10.1021/acs.est.7b04665 29318874

[15] Fetzel T, Niedertscheider M, Haberl H, Krausmann F, Erb K-H (2016) Patterns and changes of land use and land-use efficiency in Africa 1980–2005: an analysis based on the human appropriation of net primary production framework. Regional Environmental Change 16: 1507–1520.

[16] Plutzar C, Kroisleitner C, Haberl H, Fetzel T, Bulgheroni C, et al (2016) Changes in the spatial patterns of human appropriation of net primary production (HANPP) in Europe 1990–2006. Regional Environmental Change 16: 1225–1238.

[17] Huang X, Luo G, Ye F, Han Q (2018) Effects of grazing on net primary productivity, evapotranspiration and water use efficiency in the grasslands of Xinjiang, China. Journal of Arid Land 10: 588–600.

[18] Fetzel T, Gradwohl M, Erb K-H (2014) Conversion, intensification, and abandonment: A human appropriation of net primary production approach to analyze historic land-use dynamics in New Zealand 1860–2005. Ecological Economics 97: 201–208.

[19] Bin Mahbub R, Ahmed N, Rahman S, Hossain MM, Sujauddin M (2019) Human appropriation of net primary production in Bangladesh, 1700–2100. Land Use Policy 87: 4067–4067.

[20] Chen A, Li R, Wang H, He B (2015) Quantitative assessment of human appropriation of aboveground net primary production in China. Ecological Modelling 312: 54–60.

[21] Li F, Meng J (2018) Temporal and spatial variation of human appropriation of net primary productivity in the middle reaches of the Heihe River Basin. Arid Zone Research 35: 743–752.

[22] Zhang R, P. (2017) Analysis of grassland NPP and phenology in response to climate change in Xinjjiang. Lanzhou University.

[23] Han Q, Luo G, Li C, Xu W (2014) Modeling the grazing effect on dry grassland carbon cycling with Biome-BGC model. Ecological Complexity 17: 149–157.

[24] Liu L, Zhao X, Chang X, Lian J (2016) Impact of precipitation fluctuation on desert-grassland ANPP. Sustainability 8 10.3390/su8101040 28008371 PMC5170843

[25] Haberl H, Steinberger JK, Plutzar C, Erb K-H, Gaube V, et al (2012) Natural and socioeconomic determinants of the embodied human appropriation of net primary production and its relation to other resource use indicators. Ecological Indicators 23: 222–231. 10.1016/j.ecolind.2012.03.027 23470886 PMC3587410

[26] Lorel C, Plutzar C, Erb K-H, Mouchet M (2019) Linking the human appropriation of net primary productivity-based indicators, input cost and high nature value to the dimensions of land-use intensity across French agricultural landscapes. Agriculture Ecosystems & Environment 283.

[27] Xu B, Yang X, Jin Y, Wang D, Yang Z, et al (2012) Monitoring and evaluation of grassland-livestock balance in pastoral and semi-pastoral counties of China. Geographical Research 31: 1998–2006.

[28] Rong Y, Yuan F, Ma L (2014) Effectiveness of exclosures for restoring soils and vegetation degraded by overgrazing in the Junggar Basin, China. Grassland Science 60: 118–124.

[29] Han Q, Luo G, Li C, Ye H, Chen Y (2013) Modeling grassland net primary productivity and water-use efficiency along an elevational gradient of the northern Tianshan Mountains. Journal of Arid Land 5: 354–365.

[30] Han Q, Luo G, Li C, Shakir A, Wu M, et al (2016) Simulated grazing effects on carbon emission in Central Asia. Agricultural and Forest Meteorology 216: 203–214.

[31] Zhang T, Sun Y, Shi Z, Feng G (2012) Arbuscular mycorrhizal fungi can accelerate the restoration of degraded spring grassland in central Asia. Rangeland Ecology & Management 65: 426–432.

[32] Luo G, Han Q, Zhou D, Li L, Chen X, et al (2012) Moderate grazing can promote aboveground primary production of grassland under water stress. Ecological Complexity 11: 126–136.

[33] Seligman NG, Cavagnaro JB, Horno ME (1992) Simulation of defoliation effects on primary production of a warm-season, semiarid perennial-species grassland. Ecological Modelling 60: 45–61.

[34] Zhou D, Luo G, Han Q, Yin C, Li L, et al (2012) Impacts of grazing and climate change on the aboveground net primary productivity of mountainous grassland ecosystems along altitudinal gradients over the Northern Tianshan Mountains,China. Acta Ecologica Sinica 32: 81–92.

[35] Jin JX (2012) Xinjiang statistical yearbook. Beijing: China Statistics Press.

[36] Buer J, Zhao S, He F, Xu DW, Zhu XL, et al (2014) Sustainable development strategy study on Xinjiang grassland animal husbandry. Chinese Journal of Agricultural resources and regional Planning 35: 120–127.

[37] Su R, Cheng J, Chen D, Bai Y, Jin H, et al (2017) Effects of grazing on spatiotemporal variations in community structure and ecosystem function on the grasslands of Inner Mongolia, China. Scientific Reports 7 10.1038/s41598-017-00105-y 28232738 PMC5427926

[38] Baeza S, Paruelo JM (2018) Spatial and temporal variation of human appropriation of net primary production in the Rio de la Plata grasslands. Isprs Journal of Photogrammetry and Remote Sensing 145: 238–249.

[39] Wang Z, Deng X, Song W, Li Z, Chen J (2017) What is the main cause of grassland degradation? A case study of grassland ecosystem service in the middle-south Inner Mongolia. Catena 150: 100–107.

[40] Zika M, Erb K-H (2009) The global loss of net primary production resulting from human-induced soil degradation in drylands. Ecological Economics 69: 310–318.

[41] Fetzel T, Havlik P, Herrero M, Erb K-H (2017) Seasonality constraints to livestock grazing intensity. Global Change Biology 23: 1636–1647. 10.1111/gcb.13591 27976453

